# Evaluation of physical activity among adults with diabetes mellitus from Sri Lanka

**DOI:** 10.1186/1755-7682-7-15

**Published:** 2014-04-04

**Authors:** D Chathuranga Ranasinghe, Priyanga Ranasinghe, Ranil Jayawardena, David R Matthews, Prasad Katulanda

**Affiliations:** 1 Allied Health Sciences Unit, Faculty of Medicine, University of Colombo, Colombo, Sri Lanka; 2 Department of Pharmacology, Faculty of Medicine, University of Colombo, Colombo, Sri Lanka; 3 Institute of Health and Biomedical Innovation, Queensland University of Technology, Brisbane, QLD, Australia; 4 Oxford Centre for Diabetes, Endocrinology and Metabolism, University of Oxford, Oxford, UK; 5 Department of Clinical Medicine, Faculty of Medicine, University of Colombo, Sri Lanka

**Keywords:** Diabetes mellitus, Physical activity, Inactivity, Adults, Sri Lanka

## Abstract

We evaluated the patterns of physical activity (PA) and the prevalence of physical inactivity among Sri Lankan adults with diabetes mellitus. Data were collected as part of a wider cross-sectional national study on diabetes in Sri Lanka. PA during the past week was assessed using the short version of the IPAQ. Overall prevalence of physical inactivity was 13.9%. Females (3091 ± 2119) had a significantly higher mean weekly total MET minutes than males (2506 ± 2084) (p < 0.01). Inactivity of those residing in urban (17.2%) areas was higher than rural (12.6%) in all adults. Participants from Moor ethnicity were more inactive compared to others. Adults who were physically active had significantly low waist and hip circumferences, BMI and systolic blood pressure.

## Introduction

Physical inactivity has been identified as the fourth leading risk factor for global mortality [1]. Sedentary living is responsible for about one-third of deaths due to diabetes, a disease for which physical inactivity is an established risk factor [2]. The prevalence of diabetes has reached epidemic levels in Sri Lanka, in 2005 the prevalence of diabetes was 11% and 1/5^th^ of the adult population were suffering from dysglycemia (diabetes and pre-diabetes) [3,4]. There are numerous benefits of regular physical activity (PA) for patients with diabetes, such as the reduction of insulin resistance, improvement in glycaemic control and prevention of cardiovascular disease. Measuring current levels of PA is an important initial step and a public health priority. However, currently there is only limited data on PA among patients with diabetes from developing South Asian countries. The present study aims to evaluate the patterns of PA and the prevalence of physical inactivity among Sri Lankan adults with diabetes mellitus and explore its’ relationships with socio-demographic, clinical and biochemical parameters.

## Methods

Data were collected as part of a wider cross-sectional national study on diabetes conducted in Sri Lanka between August 2005 and September 2006. Detailed sampling has been previously reported [3]. Ethical approval for the study was obtained from the Ethical Review Committee, Faculty of Medicine, University of Colombo, Sri Lanka. Five thousand non-institutionalized adults >18 years of age were invited for the study. Informed written consent was taken prior to recruitment. Subjects were considered to have ‘diagnosed diabetes’ if they had been previously diagnosed at a government hospital or by a registered medical practitioner. New cases (‘undiagnosed diabetes’) were diagnosed according to the American Diabetes Association and World Health Organization criteria [5,6].

PA during the past week was assessed using the short version of the IPAQ administered by an interviewer [7]. The short IPAQ allows categorical and continuous measurements of physical activity. The continuous score allows the estimation of the weekly energy expenditure expressed in MET minutes/week (Metabolic Equivalent Task-Minutes). This is obtained by multiplying the value of energy expenditure for the given physical activity in MET by the weekly frequency (days per week) and the time in minutes (minutes per day). The categorical score classifies individual into three categories; ‘Inactive’ , ‘Moderately active’ and ‘Highly active’ [7]. An interviewer administrated questionnaire was used to obtain socio-demographic details such as age, gender, area of residence, ethnicity, level of education and household income. Additional anthropometric, biochemical and clinical details were also collected and are described elsewhere [3]. Data were analysed using SPSS version 14. In all statistical analyses *P* values <0.05 were considered significant.

## Results

Crude prevalence of diabetes mellitus was 11.9% (n = 528), with 191 new cases (36.2%). This report is based on 476 participants with diabetes after excluding those with incomplete data on PA. Mean age was 54.3 ± 12.1 years and 64.3% (n = 306) were females. Majority of the study population were Sinhalese in ethnicity (n = 398, 83.6%), from rural areas (n = 348, 73.1%). The prevalence of micro- and macro-vascular complications of diabetes in the present cohort was as follow; neuropathy – 6.6%, nephropathy – 5.7%, retinopathy – 11.9%, Stroke/TIA – 3.4% and foot ulcers – 5.1%. The overall prevalence of physical inactivity was 13.9%. The mean weekly total MET (Metabolic Equivalent of Task) minutes in the study population was 3136 ± 2059. Females (3091 ± 2119) had a significantly higher mean weekly total MET minutes than males (2506 ± 2084) (p < 0.01). The mean MET minutes of the inactive, moderately active and highly active groups were 597 ± 538, 1648 ± 883 and 4790 ± 1633 respectively.

Inactivity of those residing in urban (17.2%) areas was higher than rural (12.6%) in all adults (Table 1). Inactivity seemed to follow an increasing trend with age. Participants from Moor ethnicity were more inactive compared to other ethnicities. In all adults and in males those who only had a primary education were more active than others. Participants with highest income were more active in all adults and males, where as in females those with the highest income were the least active. Diabetes patients further burdened with concomitant hypertension and metabolic syndrome were more inactive. In all adults, those who were physically active (moderate and highly active) were significantly younger than the physically inactive (p < 0.05) (Table 2). Those who had earlier onset of diabetes seem to be more active than the others. Adults who were physically active had significantly low waist circumference and Waist to Hip Ratio. They also had lower BMI, hip circumferences and systolic blood pressure. In the highly active group, fasting blood glucose and 2 hour postprandial blood glucose were higher compared to others. The moderate and highly active groups both had higher Total and LDL cholesterol compared to the inactive group. In contrast they also had higher HDL cholesterol and lower triglycerides (Table 2).

**Table 1:** Prevalence of physical inactivity in patients with diabetes mellitus

	**Prevalence of inactivity (%)**		
	**All adults (n = 476)**	**Males (n = 170)**	**Female (n = 306)**
Area of residence			
Urban	17.2	15.1	18.7
Rural	12.6	18.8	9.5
Age			
< 30 years	0	0	0
30-39 years	8.9	10.0	8.0
40-49 years	12.0	22.0	7.1
50-59 years	17.4	20.0	16.0
60-69 years	8.3	3.2	10.8
> 70 years	24.1	31.8	18.8
Ethnicity			
Sinhalese	13.8	18.7	11.2
Tamil	0	0	0
Moor/Muslim	22.0	22.2	21.9
Level of education			
Primary education	12.0	4.5	14.0
Secondary education	14.4	19.9	11.1
Tertiary education	13.3	16.7	0
Monthly household income			
LKR < 12,000	12.3	16.3	10.8
LKR 12,000-25,000	21.2	27.9	14.3
LKR > 25,000	11.4	8.3	18.2
Central obesity			
Yes	11.4	14.8	8.3
No	16.1	22.6	14.0
Diabetes status			
Previously diagnosed	14.1	12.6	14.9
Newly diagnosed	13.5	27.1	6.3
Microvascular complications			
Neuropathy	8.6	10.7	8.0
Retinopathy	9.3	7.1	16.7
Hypertension			
Absent	11.7	17.1	8.8
Present	15.7	18.3	14.3
Metabolic syndrome			
Absent	12.2	16.1	9.0
Present	15.6	20.7	13.9

**Table 2:** Relationship between different activity groups and socio-demographic, clinical and biochemical parameters

	**Mean (±SD)**		
	**Inactive**	**Moderately active**	**Highly active**
Age	57.1 (±12.7)^a^	55.4 (±12.0)^b^	52.5 (±11.7)^a,b^
Age of diabetes onset	53.4 (±12.5)^a^	51.0 (±12.6)	48.9 (±11.9)^a^
Body mass index (kg/m^2^)	24.1 (±4.7)	23.9 (±3.9)	23.6 ( ±4.0)
Waist circumference (cm)	87.1 (±11.3)^a^	85.5 (±10.6)^b^	82.7 (±10.2)^a,b^
Hip circumference (cm)	93.9 (±10.8)	93.1 (±9.0)	92.4 (±8.8)
Waist to hip ratio	0.93 (±0.07)^a^	0.92 (±0.07)^b^	0.89 (±0.07)^a,b^
Systolic blood pressure (mmHg)	139.7 (±23.4)	136.6 (±22.0)	136.9 (±20.8)
Diastolic blood pressure (mmHg)	79.5 (±11.8)	79.6 (±11.9)	80.6 (±11.0)
Fasting blood glucose (mg/dl)	136.3 (±56.5)	136.4 (±51.1)	143.7 (±59.2)
2 h Post prandial blood glucose (mg/dl)	279.5 (±102.3)	275.3 (±92.1)	291.4 (±104.2)
Total cholesterol (mg/dl)	211.1 (±45.6)	219.1 (±45.0)	218.6 (±43.6)
LDL cholesterol (mg/dl)	132.5 (±41.4)	142.1 (±39.5)	140.9 (±39.2)
HDL cholesterol (mg/dl)	44.3 (±9.3)	46.7 (±10.1)	45.6 (±9.2)
Triglycerides (mg/dl)	171.6 (±126.8)	151.0 (±68.5)	159.9 (±89.8)

^a,b^– Mean values within a row with the same superscript are significantly different from each other (p < 0.05).

## Discussion

Prospective studies and meta-analysis support the fact that a higher level of PA is associated with lower mortality in individuals with diabetes [8]. This study reports PA patterns in a non-institutional nationwide sample of adults with diabetes from Sri Lanka. Although the benefits of PA in diabetes are well known a significant portion of Sri Lankan adult with diabetes were sedentary. Several socio-economical characteristics were associated with physical inactivity among patients with diabetes. Rural women who may be engaged in more manual occupations than the urban counterpart reported the lowest level of physical inactivity. The Moor ethnic group in Sri Lanka is considered to be at a high risk for metabolic diseases such as diabetes, obesity and metabolic syndrome. This could be partly explained by the fact that Moors with diabetes reported lowest level of PA in comparison to other ethnicities. However the IPAQ short version is not designed to differentiate various domains of the PA; therefore, robust PA measurement instruments are needed to estimate PA level and details on its distribution among patients with diabetes. Furthermore, future follow up studies to evaluate changes in physical activity patterns with time in the same cohort may help to establish whether patients’ health condition including glycaemic control and risk of complications improves/deteriorates with such change. Future studies should also focus on the reasons for sedentary behaviours among this population and applicability of culturally acceptable interventions.

## Competing interests

The authors declare they have no conflict of interests.

## Authors’ contributions

PK and DRM made substantial contribution to conception and study design. DCR, PR, RJ and PK were involved in data collection. PR, RJ, DCR, DRM and PK were involved in refining the study design, statistical analysis and drafting the manuscript. PR, RJ and PK critically revised the manuscript. All authors read and approved the final manuscript.
